# Editorial: Impact of COVID-19 pandemics and syndemics on healthcare systems worldwide

**DOI:** 10.3389/fped.2024.1523617

**Published:** 2024-11-22

**Authors:** Ozgur Karcioglu

**Affiliations:** Deptment of Emergency Medicine, University of Health Sciences, Istanbul Training and Research Hospital, Istanbul, Türkiye

**Keywords:** COVID-19, infection, pandemics, children, pediatrics, healthcare system, diagnosis, treatment

**Editorial on the Research Topic**
Impact of COVID-19 pandemics and syndemics on healthcare systems worldwide

Infectious diseases have been the leading cause of death for ages. However, pandemics are cornerstones to overwhelm the countries' healthcare systems and institutional capacities, as seen in the plague in the medieval ages, Spanish flu Asian flu in 20th century, swine flu in 2009–10 and lastly, COVID-19.

The COVID-19 pandemic has altered almost every aspect of the world, including economics, social issues, health care, and politics. Many experts consider this phenomenon to be a “syndemic”, as it is more than just an outbreak of a viral infectious disease.

The topic “Research Topic Impact of COVID-19 Pandemics and Syndemics on Healthcare Systems Worldwide” included sixteen articles addressing the effect of the COVID-19 pandemics on all of us. This Research Topic tried to cover issues related to the challenges of the pandemic disease on the human being and specifically pediatric healthcare, focusing on specific areas such as the prehospital system, emergency departments, ICUs, medical branches, preventive medicine, rehabilitation, sports medicine, and infection surveillance. This article collection evaluated studies on diagnosis and management of COVID-19 in children, COVID vaccination and related issues, geographical and socioeconomic inequalities of COVID-19 infection in children, and burden of COVID-19 in acute care worldwide.

The authorship of the articles represents the multcultural background taking care of the involved populations worldwide. This diversity comprises a great chance to enrich the discussion to create a fruitful guide to summarize viewpoints for most aspects of the pandemic era.

The work by Bermejo-Patón et al. “*The recovery and resilience plan on the long-term care system. Towards a deinstitutionalization?*” analyzed “Recovery and Resilience Plan (RRP)” to estimate the socioeconomic impact on Long-Term Care (LTC) in Spain, using the severe recession scenario triggered by COVID-19. They pointed out a substantial positive impact of RRP to mitigate the downturn in the Spanish economy following the pandemics.

The Turkish study “*Shedding light on the next pandemic path, from outpatient to ICU, the effect of vitamin D deficiency in the SARS-CoV-2 pandemic*” analyzed the role of Vitamin D on unvaccinated adults and concluded that adequate levels of Vitamin D, glucose, urea, creatinine, leucocyte, aspartate transaminase, hemoglobin, C-reactive protein, troponin, platelet/thrombocyte, ferritin, D-dimer, triglycerate, glycated haemoglobin, lactate dehydrogenase are associated with decreased likelihood to be admitted to ICU.

The study from Nakata et al. on the “*Surgical productivity recovery after the COVID-19 pandemic in Japan*” analyzed a broad range of surgical procedures and reported that surgical productivity did not appear to be affected on the long term by the COVID-19 pandemic.

The work published by Hijano et al. analyzed case investigation and contact tracing as key strategies to stop transmission of COVID-19 in a single center. They concluded that prompt implementation of these strategies are feasible and have the potential to reduce viral spread in the workplace.

The findings of the study on “*Teachers as caregivers of grieving children in school in the post-COVID-19 era*” investigated teachers' needs on childhood bereavement amidst the COVID-19 pandemic may help promote policy changes that ensure teachers' needs satisfaction in the context of pediatric grief.

The population-based descriptive study “*outcome of emergency patients transported by ambulance during the COVID-19 pandemic in Osaka Prefecture, Japan*” focused on the impact of the pandemic era on patient outcome in the prehospital setting. The authors reported an alarming finding that pandemic disease reduced the rate of ambulance calls and worsened mortality of patients transported by ambulances in Osaka.

Yang et al. studied on “*Characteristics and spectrum changes of PICU cases during the COVID-19 pandemic*” using a retrospective design. The COVID-19 pandemic has exerted a certain influence on the disease spectrum of PICU admissions, indicating a need to prioritize the respiratory, neurological, and hematological oncology systems.

An economical viewpoint was brought to the scene by the study “*The impact of COVID-19 pandemic on the world's major economies: based on a multi-country and multi-sector CGE model*” and emphasized the advancement of global anti-epidemic policies targeting economic recovery.

A multinational study by the International Consortium of Primary Care Big Data Researchers (INTRePID) investigated the “*Changes in primary care visits for respiratory illness during the COVID-19 pandemic*” in primary care settings in nine countries. They highlighted the fact that COVID-19 pandemic had a major impact on primary care visits for respiratory presentations.

In a systematic review of the literature “*Impact of the COVID-19 pandemic on access to and delivery of maternal and child healthcare services in low-and middle-income countries*” the authors reported disruption family planning services, antenatal and postnatal care coverage, and emergency and routine child services in the setting of the COVID-19 pandemic.

Zhao et al. performed a cost-effectiveness analysis from the perspective of Chinese healthcare system on influenza vaccination for heart failure patients: They suggested that adding the vaccine to standard regimens for Chinese patients with heart failure may represent a highly cost-effective option.

In an interesting multicenter cohort study, Zhang et al. analyzed the total joint arthroplasty (TJA) care patterns in China during the COVID-19 pandemic. They pointed out that patients undergoing TJA in China during the COVID-19 pandemic was associated with more severe preoperative conditions and decreased volume, costs, and readmission rates.

The study entitled “*Crucial and fragile: a multi-methods and multi-disciplinary study of cooperation in the aftermath of the COVID-19 pandemic*” by Rotondi et al. focused on the need for cooperation across nations, institutions, and individuals amidst pandemics. The work highlighted important aspects of cooperation during crises and paved the way for future explorations into cooperative decision-making.

Via a case study of COVID-19 in Taiyuan City, Guo et al. assessed “*the impact of vaccination and medical resource allocation on infectious disease outbreak management*”. They focused on the rational allocation of vaccines and medical resources to mitigate the pandemic effects and concluded that an increased maximum capacity of medical resources, will prevent the congestion and stronger resource allocation capabilities will facilitate earlier relief within a fixed total resource pool.

Ritto et al. published a study entitled “*Data-driven, cross-disciplinary collaboration: lessons learned at the largest academic health center in Latin America during the COVID-19 pandemic*” and created a set of recommended strategies to enhance collaboration within the research institution.

In brief, we should all realize that COVID-19 is not the last pandemic. It is obvious that there will be new generations and our long years to spend with masks, sanitizers, and hand disinfectants. It is important in the long run to encourage vaccination and other preventive measures, along with determining high-risk groups to provide focused protection programs to them. This kind of approach and public health perspective will be the only recipe to protect hospitals and other health institutions from being overcrowded and overwhelmed by the disastrous situation.

